# Evidence for Fgf and Wnt regulation of *Lhx2* during limb development via two limb-specific *Lhx2*-associated *cis*-regulatory modules

**DOI:** 10.3389/fcell.2025.1552716

**Published:** 2025-02-20

**Authors:** Jessica C. Britton, Anett Somogyi-Leatigaga, Billy A. Watson, Endika Haro, Cassidy G. Mulder, Kari D. Kennedy, Allen M. Cooper, Kristen L. Whitley, Ruth-Love Yeboah, Jeanyoung Kim, Micah C. Yu, Jairo D. Campos, Japhet Amoah, Shimako Kawauchi, Eunyoung Kim, Charmaine U. Pira, Kerby C. Oberg

**Affiliations:** ^1^ Department of Pathology and Human Anatomy, Loma Linda University, Loma Linda, CA, United States; ^2^ School of Medicine, Loma Linda University, Loma Linda, CA, United States; ^3^ UC Irvine Transgenic Mouse Facility, University of Irvine, Irvine, CA, United States

**Keywords:** Lhx2, vertebrate limb development, cis-regulatory module, gene regulation, FGFs, Wnt, ETS, Tcf/Lef

## Abstract

**Introduction:**

In vertebrate limb morphogenesis, wingless-related integration site (Wnt) proteins and fibroblast growth factors (Fgfs) secreted from the apical ectodermal ridge (AER) coordinate proximodistal outgrowth. Fgfs also sustain sonic hedgehog (Shh) in the zone of polarizing activity (ZPA). Shh directs anteroposterior patterning and expansion and regulates AER-*Fgfs*, establishing a positive regulatory feedback loop that is vital in sustaining limb outgrowth. The transcription factor LIM homeodomain 2 (Lhx2) is expressed in the distal mesoderm and coordinates AER and ZPA signals that control cellular proliferation, differentiation, and shaping of the developing limb. Yet how Lhx2 is transcriptionally regulated to support such functions has only been partially characterized.

**Methods/Results:**

We have identified two limb-specific *cis*-regulatory modules (CRMs) active within the *Lhx2* expression domain in the limb. Chromatin conformation analysis of the *Lhx2* locus in mouse embryonic limb bud cells predicted CRMs-*Lhx2* promoter interactions. Single-cell RNA-sequencing analysis of limb bud cells revealed co-expression of several Fgf-related *Ets* and Wnt-related *Tcf/Lef* transcripts in *Lhx2*-expressing cells. Additionally, disruption of Ets and Tcf/Lef binding sites resulted in loss of reporter-driven CRM activity. Finally, binding of β-catenin to both *Lhx2*-associated CRMs supports the associated binding of Tcf/Lef transcription factors.

**Discussion:**

These results suggest a role for Ets and Tcf/Lef transcription factors in the regulation of *Lhx2* expression through these limb-specific *Lhx2*-associated CRMs. Moreover, these CRMs provide a mechanism for Fgf and Wnt signaling to localize and maintain distal *Lhx2* expression during vertebrate limb development.

## 1 Introduction

LIM homeodomain 2 (*Lhx2*) is a transcription factor critical for regulating the development of the brain, eye, skin, and limb. In the regulation of the limb, *Lhx2* is expressed in the distal mesoderm and plays an essential role in maintaining cells in a responsive state as axis-specific signals coordinate patterned limb outgrowth (Tzchori et al., 2009; Watson et al., 2018). In *Drosophila*, *apterous* regulates dorsal fates and is required for limb outgrowth (Blair et al., 1994; Williams et al., 1991). *LHX2* is considered the vertebrate homologue of *apterous* controlling limb outgrowth (Rodriguez-Esteban et al., 1998). Knockdown of *LHX2* during limb outgrowth in chicken causes severe limb truncations (Rodriguez-Esteban et al., 1998). In mouse, another family member, *Lhx9,* is also expressed in the distal limb mesoderm and exhibits redundant functions of *Lhx2*. Knockout of both *Lhx2* and *Lhx9* disrupts limb outgrowth similar to chicken (Tzchori et al., 2009).

The loss of Lhx2/9 function disrupts several molecular pathways critical for limb patterning and outgrowth. *Fgf10* is expressed in the mesoderm of the emerging limb bud and induces the epithelium at the distal tip to thicken and form the apical ectodermal ridge (AER) (Ohuchi et al., 1997; Ohuchi et al., 1999). The AER, through Wnt proteins, secretes several fibroblast growth factors (Fgfs) that maintain *Fgf10* in the underlying mesoderm, establishing a reciprocal positive feedback loop that promotes proximodistal limb outgrowth and patterning (Ohuchi et al., 1997). In mice lacking Lhx2/9 function, *Fgf10* expression is greatly reduced (Tzchori et al., 2009).

Lhx2 also regulates sonic hedgehog (Shh) which mediates anteroposterior patterning and outgrowth. Shh is secreted from the zone of polarizing activity (ZPA), a collection of mesodermal cells in the distal posterior limb bud (Yang et al., 1997; Zhu et al., 2008). Shh also supports AER *Fgfs* expression, and in turn, AER-Fgfs maintain the ZPA’s expression of *Shh* in another reciprocal positive feedback loop (Niswander et al., 1994). In the absence of Lhx2/9 function, *Shh* expression is markedly decreased (Tzchori et al., 2009).

Although Lhx2 is critical for proximodistal and anteroposterior patterning, the mechanisms that regulate Lhx2 for these important roles have not been characterized. *Cis*-regulatory modules (CRMs) control spatiotemporal gene expression (Kolovos et al., 2012; Lewis et al., 2019) and thus were the focus of this investigation. In a previous study, Lee and colleagues (Lee et al., 2011) identified four CRMs labeled as conserved non-coding DNA elements (CNEs) within the *Lhx2* locus that were active in the central nervous system overlapping *Lhx2* expression in mice. However, none of the CNEs showed activity in the limb.

In this study, we used vertebrate conservation combined with mouse limb chromatin modification data to examine and identify potential limb-associated CRMs within the *Lhx2* locus. We utilized the chicken enhancer bioassay (Oberg et al., 2002; Pira et al., 2008; Uchikawa et al., 2003) to screen and localize functional CRMs in the limb *in ovo* and confirmed their pattern of activity in transgenic mice. We analyzed the co-expression of candidate transcription factors in *Lhx2-*expressing murine limb cells and identified transcription factor binding site sequences important for CRM activity. Our collective results suggest a mechanism by which key transcription factors linked to Fgf and Wnt signaling regulate *Lhx2* expression through limb-specific CRMs during limb development.

## 2 Materials and methods

### 2.1 Prediction of *cis*-regulatory modules

Conserved non-coding DNA sequences 1 megabase (Mb) both upstream and downstream of the *Lhx2* gene were identified by pairwise alignment using VISTA Genome browser (http://genome.lbl.gov/vista/), UCSC Genome browser (https://genome.ucsc.edu), and the enhancer prediction database Enhancer Atlas (http://www.enhanceratlas.org/). Sequences exhibiting at least 70% homology between vertebrate species and possessing binding sites for limb-associated regulated transcription factors were selected. We excluded conserved regions described by Lee et al. (2011) since none of these were found to be active in limbs. Available embryonic day 11.5 (E11.5) and E12.5 mouse limb ChIP-seq data were used to determine enhancer-related chromatin modification proteins coincident with predicted conserved regions (Gene Expression Omnibus database (GEO; http://www.ncbi.nlm.nih.gov/geo/) under accession numbers: GSE42413 for H3K27ac (Cotney et al., 2013); GSE42237 for H3K4me2 and H3K27me3 (DeMare et al., 2013); GSE13845 for p300 (Visel et al., 2009); RNAP2 and Med12 (Berlivet et al., 2013)).

Conserved regions were scored based on the number of aligned marks, and regions associated with 3 or more enhancer-related chromatin marks were chosen as candidates for potential *cis*-regulatory modules (PCRMs) (Supplementary Table S1). Regions characterized by two or less active regulatory marks or by repressive regulatory marks were not selected for further analysis. PCRMs were between 150 and 2,100 base pairs in length and were isolated from chicken genomic DNA by PCR using the primers listed in Supplementary Table S2. PCRM DNA sequences were cloned into pCRII TOPO vector (Thermofisher Scientific, Waltham, MA; Catalog #K450002) and then sub-cloned into a thymidine kinase (tk) promoter-driven enhanced GFP (eGFP) reporter construct (generous gift of Masanori Uchikawa) (Uchikawa et al., 2003) for assessment *in ovo*.

### 2.2 Chromatin conformation and accessibility

Merged hic reads from E11.5 mouse limb Hi-C data ((Kraft et al., 2019); under accession number GSE116794) were converted into the cooler (hic.cool) format using hic2cool converter package (https://github.com/4dn-dcic/hic2cool). Using the Hi-C Explorer software (http://hicexplorer.readthedocs.io/) (Ramírez et al., 2018; Wolff et al., 2018), TAD calling was performed using the hicFindTAD command with the following parameters: threshold value of 0.05 and delta value of 0.01 and a min and max depth of 3,000 and 31,500, respectively. The output file (domains.bed) was used to plot TAD boundaries (Supplementary Table S3) and visualized using pyGenome tracks (Lopez-Delisle et al., 2021). E11.5 mouse limb CTCF ChIP-seq data was obtained from DeMare and colleagues ((DeMare et al., 2013); under accession GSE42237), and visualized using UCSC Genome Browser (https://genome.ucsc.edu). Accessibility of the *Lhx2* locus was evaluated using E11.5 mouse ATAC-seq data reported by Jhanwar and coworkers ((Jhanwar et al., 2021); under accession number GSE164736).

### 2.3 CRM activity in the chicken limb bioassay

Chicken eggs (Chino Valley Ranchers, Colton, CA) were incubated in a humidified chamber according to Hamburger and Hamilton (HH) stages (Hamburger and Hamilton, 1951). To screen for potential CRM activity, we performed confined microelectroporation (CMEP) to transfect the distal chicken limb bud mesoderm at HH23 modified from the technique described by Oberg et al. (2002). A cocktail consisting of 2 μg/μL of PCRM-eGFP reporter constructs, 0.2 μg/μL pCAGGS-RFP, 0.25% fast green, and Tris-EDTA (TE) buffer was injected into the limb mesoderm ∼50 μm from distal tip. The pCAGGS-RFP is a β-actin promoter-driven RFP plasmid that was used to assess transfection efficiency (kind gift from Cheryll Tickle). The insulated anode needle was inserted into the limb mesoderm anterior to the DNA injection site, whereas the blunt cathode was placed posterior to the injection site, touching the tip of the distal limb bud. Electroporation was performed using the CUY21 Electroporator (Protech International, Boerne, TX) with the following parameters: 10 pulses of 30 V at a duration of 25 ms and with 50 ms intervals. Embryos were incubated in a humidified chamber at 37°C for 24 h and then harvested. Fluorescence was visualized and digital images were acquired using the Sony DKC-5000 or the Leica MZ 10F dissecting microscope camera. Images were compiled in Adobe Photoshop Version 2024.

To determine the pattern of CRM activity, we performed targeted regional electroporation (TREP) to transfect CRM-eGFP constructs into the presumptive limb bud at HH14, as described by Pira et al. (2008). Embryos were incubated in a humidified chamber at 37°C for 48 h and then harvested. Fluorescence was visualized and digital images were acquired as described above.

### 2.4 CRM activity in transgenic mice

The mouse DNA sequence of *CRM* *(−8)/LADLRM* and *CRM* *(−9)/LASARM* were cloned into the PCR4-Shh-lacZ-H11 reporter (Addgene plasmid # 139098; http://n2t.net/addgene:139098) via Gibson Assembly (New England Biolabs, Ipswich, MA; Catalog# E2611S). The constructs were used to generate transgenic mice embryos (University of California Irvine Transgenic Mouse Facility, Irvine, CA). Embryos were harvested at E11.5 and processed for detection of LacZ activity as described by Shen et al. (2017). CRMs that showed reproducible expression patterns in at least three embryos were designated enhancers.

### 2.5 Fluorescence imaging quantification

For each image, red and green fluorescence channels of images were separated and converted to grayscale (16 bit) using the image analysis software FIJI (Schindelin et al., 2012; Shihan et al., 2021). The region of interest was outlined based on the selected threshold and the mean fluorescence intensity calculated from the measured mean of both RFP and GFP images. GFP fluorescence intensity was normalized to that of RFP. One-way ANOVA followed by Dunnett’s multiple comparison test with alpha = 0.05 was performed using GraphPad Prism (version 10.0) to determine difference in the mean fluorescence intensity. Data were summarized using box and whisker plots showing the interquartile range, range, and median values, with p-value format as: *: p < 0.05, ns: non-significant.

### 2.6 Whole mount *in situ* hybridization

Chicken cDNA *LHX2* and *SHH* were sub-cloned into pCRII-TOPO vector following the manufacturer’s instructions and used to generate digoxigenin-labeled mRNA probes (Yamada et al., 1999). Whole mount *in situ* hybridization was performed on HH23 chicken embryos as described (Watson et al., 2018). Embryos were fixed in MEMFA (0.1 M MOPS, 2 mM EGTA, 1 mM MgSO4, 3.7% Formaldehyde) for 90 min at room temperature or overnight at 4°C and then dehydrated in 90% methanol. Proteinase K treatment was 10 μg/mL with an incubation time of 7 min at room temperature. Hybridization and post-hybridization washes were carried out at 60°C and 63°C, respectively. At least 5 embryos per gene were analyzed.

### 2.7 Site-directed mutagenesis of CRM-reporter constructs

Putative transcription factor binding sites with prediction binding scores of p < 10^−2^ were identified and visualized using the JASPAR Transcription Factor Binding Site 2024 Database track on UCSC Genome browser (https://genome.ucsc.edu). Primers to the core nucleotide sequences of binding sites were designed to introduce a restriction enzyme to disrupt the sequence (Supplementary Table S4). The primers containing the mutation core sequences were introduced into the chicken *CRM (-8)/LADLRM*, *CRM (-9)/LASARM*-eGFP, and *ZRS*-eGFP reporter constructs via the QuickChange Lightning Site-Directed Mutagenesis Kit (Agilent Technologies, Santa Clara, CA, Catalog# 210513) following manufacturer recommendations. The mutated constructs were sequence-validated and rechecked on Ciiider, a tool for predicting transcription factor binding sites, to determine if new putative transcription factor binding sites were generated by the mutation (Supplementary Figure S1).

### 2.8 Analysis of publicly available single-cell RNA-seq data

E11 mouse forelimb mesoderm single-cell RNA sequencing (scRNA-seq) data reported by He and colleagues (He et al., 2020) was evaluated for cells expressing *Lhx2*. Using Partek^®^ Flow^®^ software, v10.0.23.0214 (RRID:SCR_011860), filtered h5 matrices were imported and processed as described (Yeboah et al., 2023). In brief, batch correction was used to minimize cross-sample variation and differential expression between *Lhx2*-expressing (*Lhx2*+) and *Lhx2* non-expressing (*Lhx2-)* cells (normalized expression lower than 0.5) using ANOVA and Hurdle. Principal component analysis (PCA) was conducted for dimensionality reduction and visualization of relationships among sequenced cells. The number of cells expressing each transcript of interest was recorded (Supplementary Table S5), and t-SNE plots were generated to visualize and analyze co-segregation of transcripts of interest with *Lhx2*+ cells. We also confirmed the mesodermal enrichment of cell data analyzed (Supplementary Figure S2).

## 3 Results

### 3.1 Two *Lhx2*-associated *cis*-regulatory modules are active within the *Lhx2* expression domain during limb development

**FIGURE 1:**
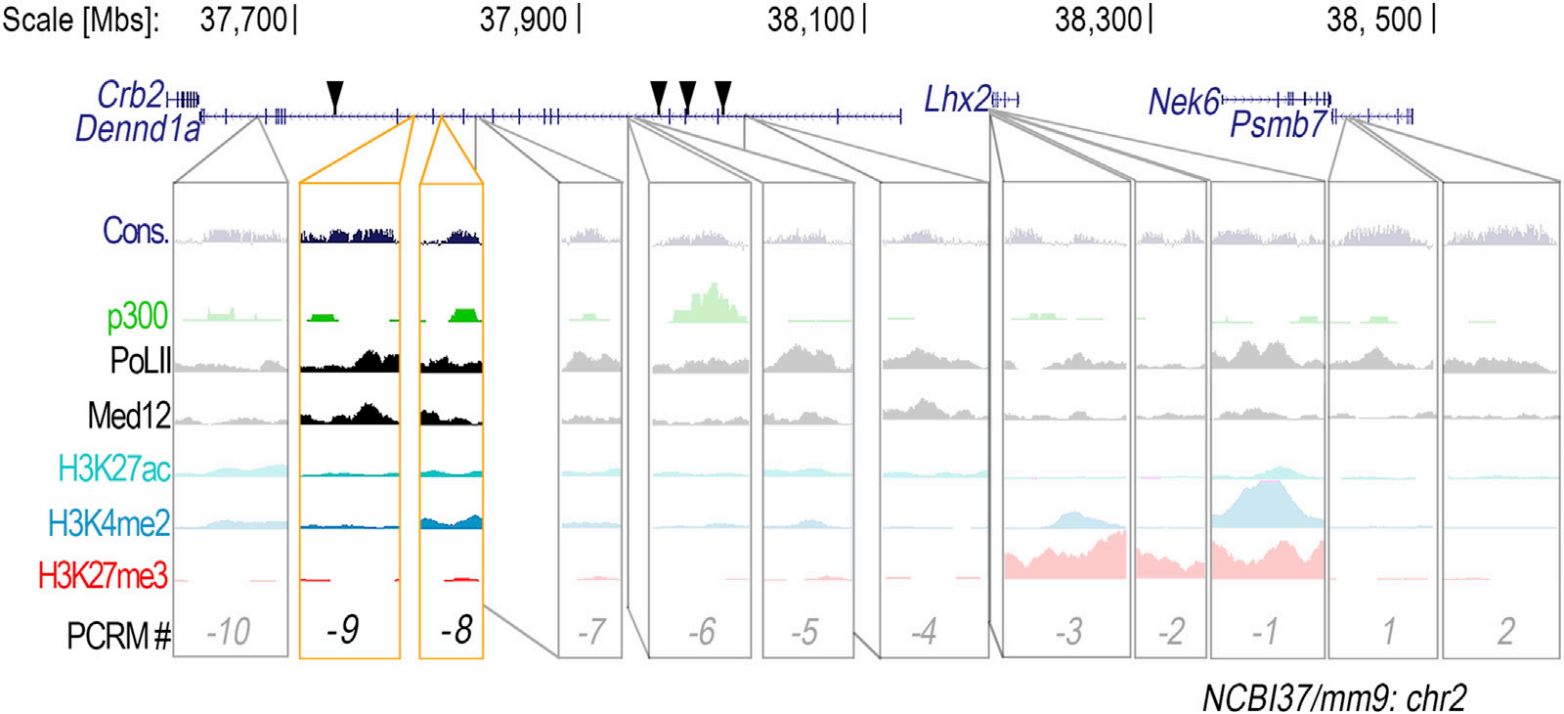
Twelve conserved non-coding DNA regions within the *Lhx2* locus are suitable candidates for potential *cis*-regulatory modules. Conserved (Cons.) non-coding DNA regions in the *Lhx2* locus were found to be associated with enhancer-related chromatin modification marks–H3K4me2, H3K27ac, p300, RNAP2, and Med12 – and thus potential *cis*-regulatory modules (PCRMs). PCRMs located upstream of the *Lhx2* promoter were given a negative number and PCRMs located downstream were given a positive number. Location of active CNEs described by Lee and colleagues (Lee et al., 2011) are marked by black triangles. *CRM (-8)* and *CRM (-9)*, located within the *Dennd1a* gene, are highlighted in yellow since they showed enhancer activity in the distal limb.

*In silico* analysis of the *Lhx2* locus revealed a total of 49 conserved non-coding DNA sequences with 70% or more sequence homology between vertebrate species both upstream and downstream of the *Lhx2* promoter. These 49 conserved regions also possessed binding sites for limb-associated transcription factors (Supplementary Table S1). Twelve out of the 49 conserved regions were associated with three or more enhancer-related chromatin marks: H3K27ac, H3K4me2, p300, RNAP2, and Med12 (Figure 1), indicating potential *cis*-regulatory function. Ten of the potential *cis*-regulatory modules (PCRMs) are located upstream of the *Lhx2* promoter (PCRM *(−1)* through *(−10)*, while two are located downstream (PCRM *1* and *2*) (Figure 1).

Regulation of gene expression by long-range CRMs (>5 kb from the promoter) requires chromatin folding that facilitates CRM-promoter interactions (Kraft et al., 2019; Long et al., 2022). Frequently interacting chromatin regions called topologically associated domains (TADs) are bordered by sequences that bind CCCTC-binding factors (CTCF) that define TAD boundaries within which chromatin interactions are most likely to occur (Kraft et al., 2019; Long et al., 2022). In addition, accessible chromatin is needed for CRMs to be functionally active (Buenrostro et al., 2013; Buenrostro et al., 2015). Therefore, we analyzed the chromatin organization and accessibility of the *Lhx2* locus using published Hi-C data from embryonic day 11.5 (E11.5) limbs (Kraft et al., 2019), CTCF ChIP-seq (DeMare et al., 2013), and ATAC-seq data (Jhanwar et al., 2021).

**FIGURE 2:**
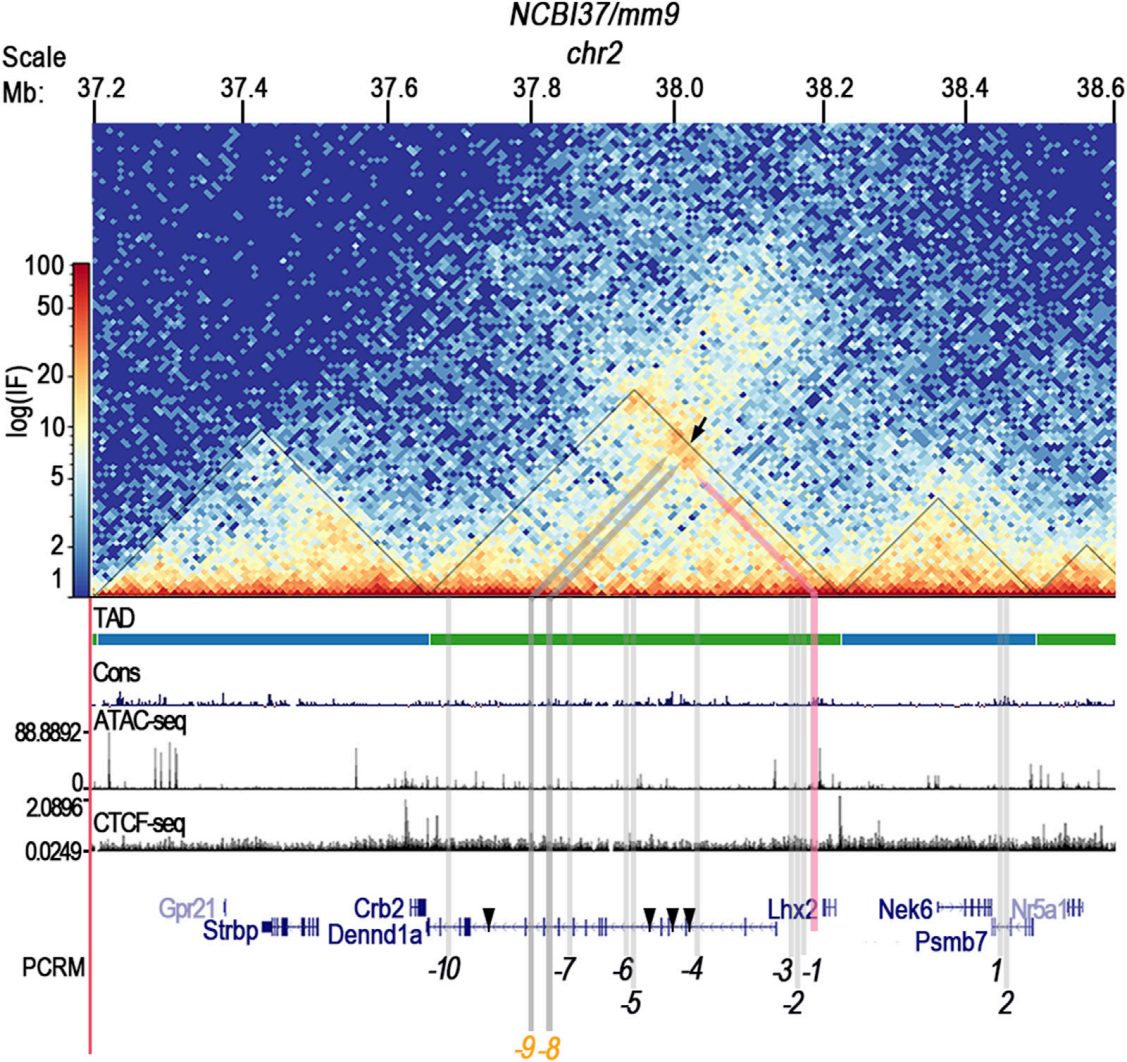
*CRM (-8)* and *CRM (-9)* are predicted to interact with the *Lhx2* promoter and are accessible for gene regulation. Hi-C heatmap of mouse E11.5 limb buds depicts regions of interaction within the *Lhx2* locus. Color bar represents the log of interaction frequency (log(IF). Black triangles highlight the border of topologically associated domain (TAD) interactions, and TAD boundaries are defined by peak calling from CTCF ChIP-seq data aligned beneath the Hi-C data. *CRM (-8)* and *CRM (-9)* are located in intron 11 and 12, respectively, of the *Dennd1a* gene (gray highlight) and are predicted to interact with the *Lhx2* promoter (black arrow) within the same TAD. The pink arrow highlights the *Lhx2* promoter. Aligned ATAC-seq data shows that the chromatin within this TAD is in an open state for transcription.

*Lhx2* and the *Dennd1a* gene, wherein many of the upstream PCRMs reside, are flanked by two CTCF binding sites signifying that they are both within the same TAD (Figure 2). Hi-C data identified strong interactions between the *Lhx2* promoter and several PCRMs including −*5*, −*7*, −*8*, −*9*, and −*10*. ATAC-seq in the E11.5 limbs demonstrated accessibility of the PCRMs *-7, -8, -9*, and *-10* (Figure 2) consistent with functional activity.

**FIGURE 3:**
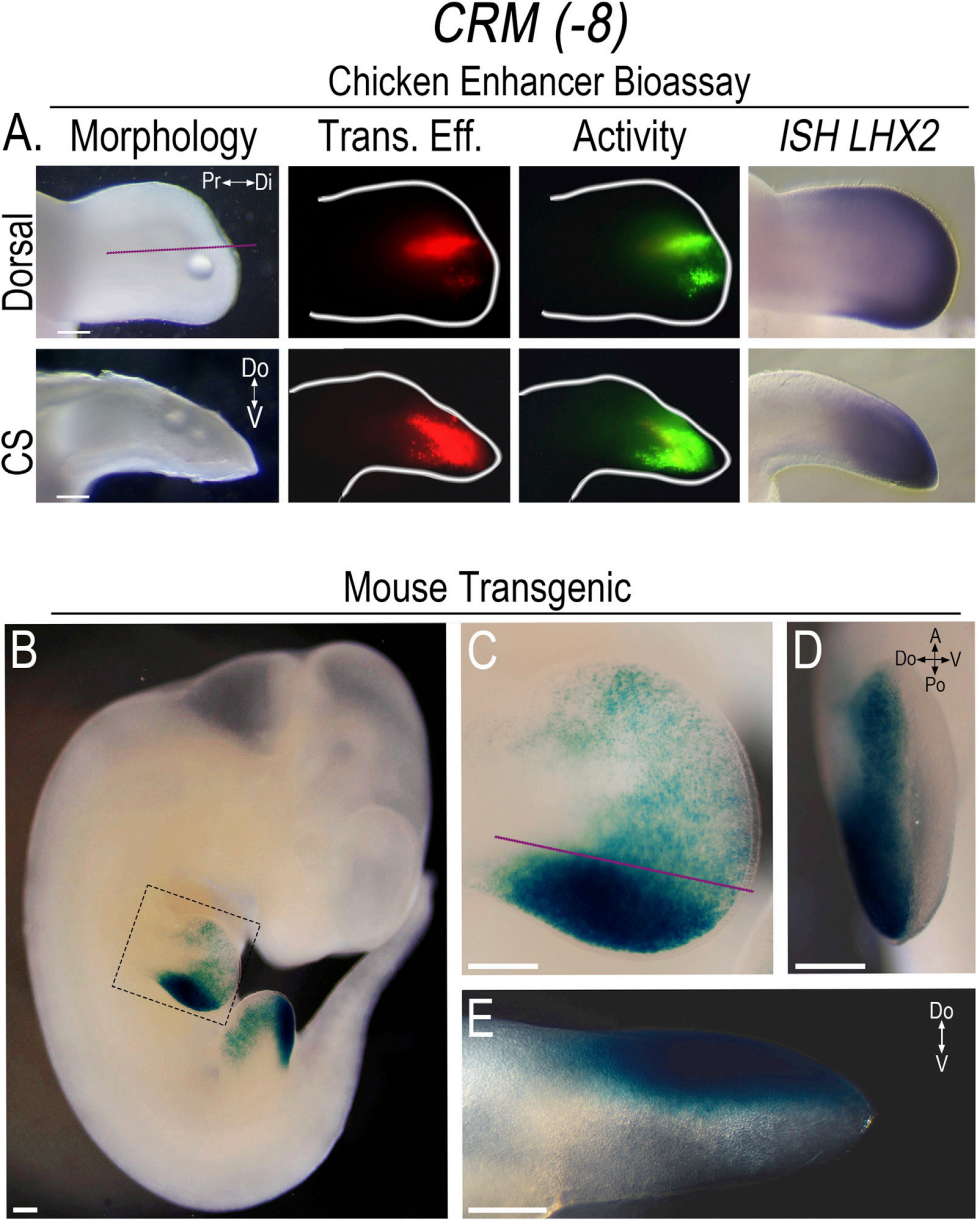
*CRM (-8)* shows robust activity in the distal limb overlapping *Lhx2* expression. **(A)** Dorsal view of limb bud shows the activity (Activity) of *CRM (-8)* (n = 31) in the distal limb mesoderm overlapping *LHX2* expression (*ISH LHX2*), using the chicken enhancer bioassay. Purple lines indicate longitudinal cross-sections (CS) of the limb bud shown in the bottom row. Note: *CRM* *(−8)* exhibits activity in both the dorsal and ventral mesoderm. Transfection efficiency (Trans. Eff) was determined with a *β*-actin promoter-driven RFP plasmid (the small bubble present in each limb bud indicates mineral oil used to seal the DNA cocktail into the mesoderm at the injection site). **(B)** LacZ staining showing the activity pattern of *CRM (-8)* (n = 5) in E11.5 transgenic mouse embryos. **(C)** Close-up of the forelimb (highlighted by the dotted box in “**(B)**”) showing the broad distal limb activity with accentuated activity over the ZPA. **(D)** End-on view of the forelimb showing activity restricted to the dorsal mesoderm. **(E)** Longitudinal cross-section of the forelimb in “**(C)**” demonstrating the dorsally restricted activity. Scale bars: 250 µm. Abbreviations used: A (Anterior), Po (Posterior), Pr (Proximal), Di (Distal), V (Ventral), Do (Dorsal).

**FIGURE 4:**
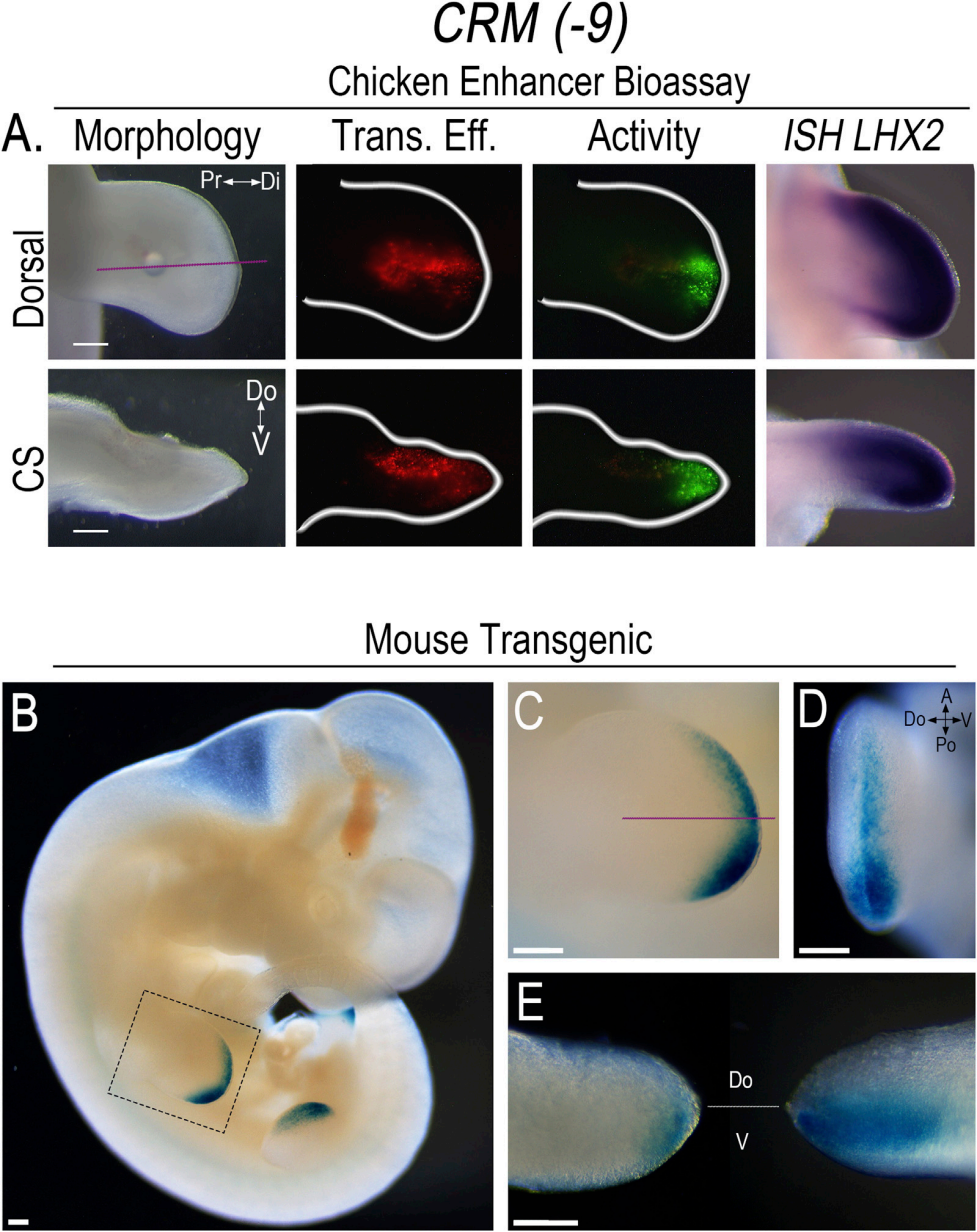
*CRM (-9)* exhibits activity in the distal sub-AER mesoderm overlapping the domain of *Lhx2* expression. **(A)** Dorsal view of limb bud shows the activity (Activity) of *CRM (-9)* (n = 30) in the distal sub-AER mesoderm overlapping *LHX2* expression (*ISH LHX2*), using the chicken enhancer bioassay. Purple lines indicate longitudinal cross-sections (CS) of the limb bud shown in the bottom row. Transfection efficiency (Trans. Eff) was determined with a *β*-actin promoter-driven RFP plasmid. The small bubble present in each limb bud indicates mineral oil used to seal the DNA cocktail into the mesoderm at the injection site. **(B)** LacZ staining showing the activity pattern of *CRM (-9)* (n = 11) in E11.5 transgenic mouse embryos. **(C)** Close-up of the forelimb (highlighted by the dotted box in “**(B)**”) showing the distal sub-AER activity that is accentuated posteriorly. **(D)** End-on view of the forelimb showing activity in both the dorsal and ventral mesoderm. **(E)** Longitudinal cross-section of the forelimb in “**(C)**” viewed towards the anterior aspect (left) shows activity accentuated in the ventral sub-AER mesoderm, and the posterior aspect (right) shows activity in both the dorsal and ventral mesoderm. Scale bars: 250 µm. Abbreviations used: A (Anterior), Po (Posterior), Pr (Proximal), Di (Distal), V (Ventral), Do (Dorsal).

To screen the PCRMs for enhancer activity, we isolated the chicken sequences using PCR, constructed CRM-eGFP reporters, and then used confined microelectroporation (CMEP) (Oberg et al., 2002) to focally transfect the CRM constructs into the distal limb mesoderm of Hamburger Hamilton stage 23 (HH23) chicken wing buds. Using this chicken limb bioassay, two of the twelve PCRMs we demonstrated were actual CRMs, (*CRM (-8)* (191 bp sequence) and *(-9)* (684 bp sequence)). They showed robust, consistent activity in the distal limb mesoderm overlapping the *LHX2* expression domain (Figures 3, 4). *CRM (-8)* was active in a broad distal band of limb mesoderm, extending about 500 μm from the tip and was present in both the dorsal and ventral mesoderm (Figure 3A). *CRM (-9)* had activity confined to the distal 100 μm of the mesenchyme abutting the apical ectodermal ridge (AER) (Figure 4A), with symmetrical dorsal and ventral activity. *CRM (-7)* exhibited weak, inconsistent activity in the distal limb bud (n = 10/45, Supplementary Figure S3A) and was not evaluated further.

The corresponding murine sequences for *CRM (−8)* and *CRM (-9)* were also isolated and screened in the chicken limb bioassay using CMEP to confirm that they exhibited the same distribution of activity. To better demonstrate the pattern of CRM activity, we used the murine sequences to generate *CRM*-Shh-lacZ-H11 reporter constructs for transient transfection.

In embryonic day 11.5 (E11.5) transgenic mice, the activity pattern of *CRM (-8)* showed a broad rim of activity that extended about 500 μm from the distal tip covering much of the distal mesoderm. However, the intensity of activity varied, with scattered, punctate activity in the anterior limb mesoderm and markedly enhanced activity in the posterior mesoderm overlapping the zone of polarizing activity (ZPA) domain (Figures 3B, C). A noted difference in the activity pattern of *CRM (-8)* in the transgenic mice was the sharp restriction of activity to just the dorsal compartment (Figures 3D, E). To determine whether the same pattern of activity was also present in the chicken model, we performed targeted regional electroporation (TREP) of the murine *CRM (-8)*-eGFP reporter into the presumptive limb mesoderm prior to limb bud formation, at HH14. The murine *CRM (-8)* showed the same anteroposterior pattern of activity in the chicken limb as seen in the mouse with accentuated activity in the posterior limb mesoderm. However, in contrast to the mouse model, activity was present in both dorsal and ventral mesoderm (Supplementary Figure S3B). These data indicate that the chicken ventral limb mesoderm can activate murine *CRM (-8),* while the mouse either lacks this capacity or is suppressing activity. Further work is needed to investigate this species-specific difference in ventral limb mesoderm.

The pattern of *CRM (-9)* activity in E11.5 transgenic mouse embryos was confined to the distal sub-AER mesoderm, with expanded activity in the posterior distal sub-AER region (Figures 4B–E). The posteriorly expanded *CRM (-9)* activity was present in both dorsal and ventral mesoderm, but was increased in the ventral mesoderm. In the anterior half, activity was even more accentuated ventrally (Figure 4E). This data, like that of *CRM (-8),* also shows a dorsoventral asymmetry that was not present in the chicken limb model (Figure 4A) and worthy of further investigation.

Neither *CRM (-8)* nor *(-9)* exhibited activity in other *Lhx2* expression domains in the transgenic mice indicating that the activity was limb-specific. With extended staining times, some weak reporter staining was observed in the basal plate of the developing spinal cord (Supplementary Figure S3C); however, *Lhx2* is not expressed in this region (Lee et al., 2011). Thus, we considered this as non-specific staining or low-level construct-related activation. Since the activity of both CRMs are limb-specific, overlap *Lhx2* expression, and are predicted to interact with the *Lhx2* promoter, we renamed these CRMs based on their pattern of activity as follows: *CRM (-8)* as *Lhx2*-associated distal limb regulatory module (*LADLRM*) and *CRM (-9)* as *Lhx2*-associated sub-AER regulatory module (*LASARM*).

### 3.2 *Ets* and *Tcf/Lef* transcripts are co-expressed in *Lhx2+* mouse limb cells

Fgfs and Wnts are known to work together to coordinate limb outgrowth and AER initiation and formation (ten Berge et al., 2008). Ets transcription factor proteins relay FGF signaling via the Ras/MAPK pathway to regulate limb outgrowth and patterning (proximodistal and anteroposterior) (Sharrocks, 2001; Yordy and Muise-Helmericks, 2000; Mao et al., 2009; Yamamoto-Shiraishi et al., 2014). In addition, Tcf/Lef transcription factor proteins are downstream effectors of Wnt/β-catenin signaling and regulate limb morphogenesis, patterning, and AER maturation (Galceran et al., 1999; Kardon et al., 2003). Such roles for Ets and Tcf/Lef proteins during limb development suggest that these transcription factors may regulate *Lhx2* expression.

Several *Ets* and *Tcf/Lef* transcripts are expressed in mouse limbs (Galceran et al., 1999; Lettice et al., 2012; Mathew et al., 2011; Wu et al., 2012; Yamamoto-Shiraishi et al., 2014). To determine whether Ets and Tcf/Lef transcription factors are present in *Lhx2-*expressing (*Lhx2+)* cells and thus capable of its regulation, we analyzed published mouse forelimb mesoderm single-cell RNA-sequencing (scRNA-seq) data (He et al., 2020). *Msx1* and *Wnt5a* were used as makers for the distal sub-AER cells, and *Meis2* was used as a marker for proximal limb mesoderm (Galceran et al., 1999; Zhang et al., 2023).

Principal component analysis (PCA), displayed as t-SNE plots, demonstrated co-segregation of *Lhx2*+ cells with *Msx1+* and *Wnt5a+* cells consistent with distal limb mesenchyme. In contrast, most *Meis2+* cells marking the proximal limb mesoderm do not co-segregate with *Lhx2+* cells. A fraction of the *Meis2+* cells, however, do overlap with *Lhx2*+ cells, congruent with an early stage of limb development before limb elongation fully separates proximal from distal.

**FIGURE 5:**
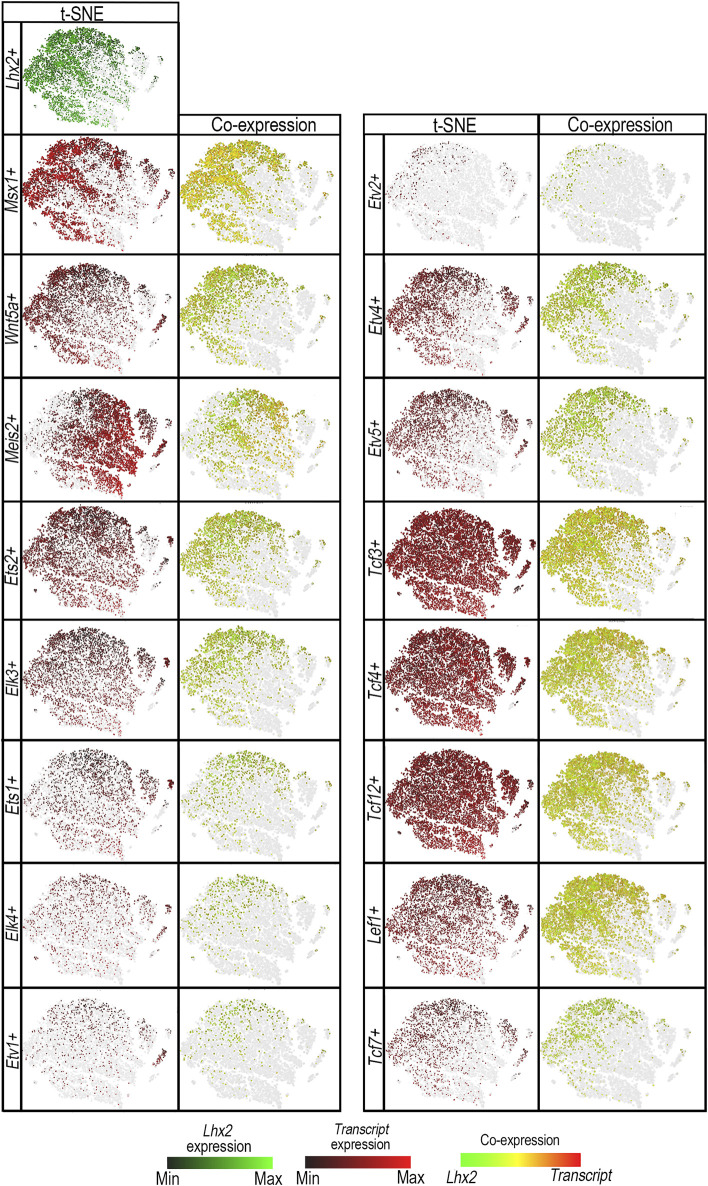
ETS and TCF/LEF transcription factor proteins are co-expressed in *Lhx2*+ cells. t-distributed stochastic neighbor embedding (t-SNE) plots shows segregation of E11.0 limb bud mesodermal cell populations by principal component analysis in *Lhx2*-expressing (*Lhx2+*) cells (green) vs. *Ets* transcripts: *Ets1*+, *Ets2*+, *Elk3+, Elk4+, Etv1+, Etv2+, Etv4*+, or *Etv5+* cells (red), and *Tcf*/Lef transcripts: *Tcf3*+, *Tcf4* +, *Tcf12+*, *Tcf7+,* or *Lef1*+ cells (red) in scRNA-seq E11 mouse limb data. The levels of *Lhx2* expression range from bright green (high) to black (low), while candidate transcription factor expression levels range from red (high) to black (low). Cells expressing both *Lhx2* and the candidate transcript are illustrated on a range from green to red, with yellow indicating similar levels of expression. The gray represents cells that do not express candidate transcripts, but are shown to preserve the segregation clusters and shape of the plot. *Msx1+* and *Wnt5a +* cells were used as markers for the distal mesoderm and showed co-expression with *Lhx2+* cells (yellow/orange) and co-segregation in the t-SNE plots with *Lhx2+* cell populations. *Meis2+* cells indicate the proximal mesoderm and do not co-segregate with *Lhx2+* cells. *Ets1*+, *Ets2*+, *Elk3+, Elk4+*, and *Etv1+* cells are ubiquitous throughout the limb bud with some cells segregating with *Lhx2+* cells in the distal limb mesoderm and co-expressing *Lhx2* (yellow/orange). Similarly, *Tcf3*+, *Tcf4* +, *Tcf12+*, and *Lef1*+ cells are present in most limb bud cells, but cells that do co-segregate with *Lhx2+* cells also co-express *Lhx2*. *Etv2+, Etv4*+, *Etv5+*, and *Tcf7+* cells display a similar segregation pattern with *Lhx2+* cells with most cells co-expressing *Lhx2*.

Multiple *Ets* transcripts (*Ets1, Ets2, Elk3, Elk4*, *Etv1, Etv2,* *Etv4*, and *Etv5*) are co-expressed in *Lhx2+* murine (Figure 5) and human limb cells (Supplementary Figure S4). *Ets2*, *Elk3*, *Ets1*, *Elk4*, and *Etv1* are ubiquitously expressed in limb cells (Figure 5). However, there are more cells expressing *Ets2*, *Elk3*, and *Ets1* transcripts than *Elk4* and *Etv1* (Supplementary Table S5). A portion of the cells that segregate with *Lhx2+* cells by PCA also co-express *Lhx2*. *Etv4*, *Etv5*, and *Etv2* expressing cells share a closer principal component segregation pattern with *Lhx2+* cells (Figure 5), and most of those cells co-express *Lhx2* (Supplementary Table S5).

Similarly, multiple *Tcf/Lef* transcripts (*Tcf3*, *Tcf4*, *Tcf7*, *Tcf12,* and *Lef1)* are co-expressed in *Lhx2+* murine (Figure 5) and human limb cells (Supplementary Figure S4). Cells expressing *Tcf3*, *Tcf4*, *Tcf12*, and *Lef1* transcripts segregate ubiquitously throughout limb cells with a fraction of them co-expressing *Lhx2*. *Tcf7*+ cells appear to segregate with *Lhx2+* cells, and although *Tcf7* is expressed in fewer cells (Supplementary Table S5) than other *Tcf/Lef1* transcripts (*Tcf3*, *Tcf4*, *Tcf12*, *Lef1*), most *Tcf7*+ cells co-express *Lhx2* (Figure 5).

Since *Etv4+*, *Etv5+*, *Etv2+*, *Tcf7+,* and *Lef1*+ cells co-express *Lhx2* and demonstrate co-segregation patterns most similar to *Lhx2+* cells, we conclude that these transcription factors are the best candidates for regulating *Lhx2* expression via *LADLRM* and *LASARM*.

### 3.3 Ets and Tcf/Lef transcription factor binding sites are critical for *CRM (−8)/LADLRM* and *CRM (−9)/LASARM* activity

**FIGURE 6:**
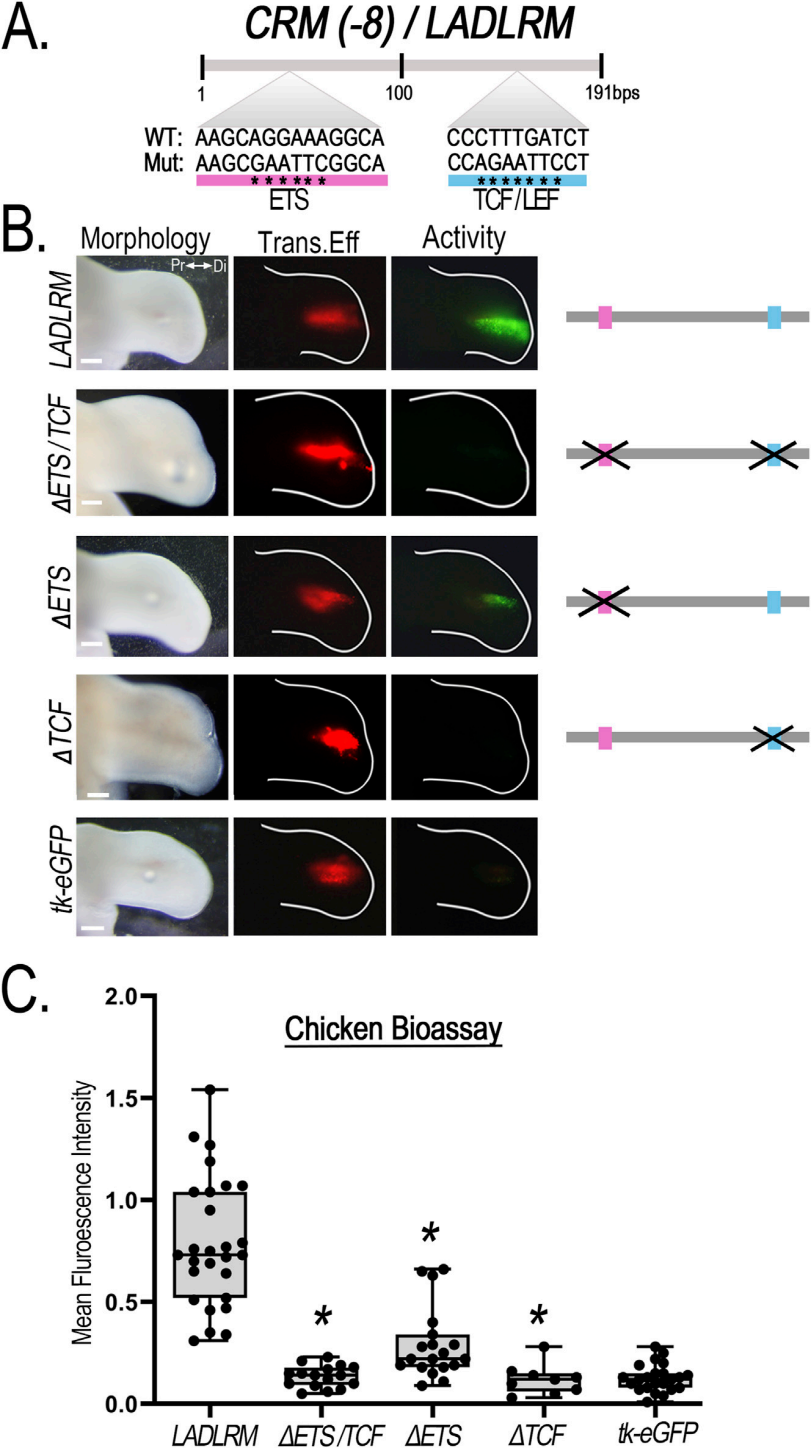
Disruption of a single putative TCF/LEF binding site removes *CRM (-8)*/*LADLRM* enhancer activity. **(A)** Schematic representation of the relative location of the putative ETS (pink) and TCF/LEF (blue) transcription factor binding site sequence in *LADLRM*. Asterisks (*) indicate the nucleotide base pair substitutions performed to generate mutated (Mut) sequences compared to the wild type (WT). **(B)** Simultaneous disruption of putative ETS and TCF binding site eliminates *LADLRM* activity. Alteration of predicted ETS binding site (ΔETS, n = 19) significantly reduces activity, while simultaneous disruption of the ETS and TCF/LEF binding site (ΔETS/TCF, n = 16) or loss of the TCF/LEF binding site (ΔTCF, n = 9) alone removes *LADLRM* activity to the level of the empty reporter vector (tk-eGFP, n = 15). **(C)** Boxplots depicting the mean fluorescence intensity of the CRM-GFP (normalized to RFP) using Fiji software. One-way ANOVA followed by Dunnett’s multiple comparison test was performed using *LADLRM* (n = 28) as the positive control *p < 0.05. Abbreviations used: Pr (proximal), Di (distal). Scale bars: 250 µm.

JASPAR transcription factor binding site prediction database identified one putative Ets and one Tcf/Lef binding site within *LADLRM* that is conserved among human, mouse, and chicken with prediction binding scores of p < 10^−2^ (Figure 6; Supplementary Figure S5). To determine whether these binding sites are important for enhancer activity, we performed site-directed mutagenesis of the core Ets nucleotide sequence 5′-GGAA-3′ (Cooper et al., 2015; Sharrocks, 2001) and Tcf/Lef core nucleotide sequence 5′-SCTTTGATS-3′ (Cadigan and Waterman, 2012) in the chicken *LADLRM* sequence. Disruption of both ETS and TCF/LEF binding sites simultaneously (ΔETS/TCF) abolished enhancer activity. Mutation of the ETS binding site (ΔETS) significantly reduced enhancer activity (Figure 6) compared to wildtype, but was insufficient to remove activity. On the other hand, mutation of TCF/LEF binding site (ΔTCF) was sufficient to eliminate activity to negative control levels (empty reporter vector) (graph to negative control in Supplementary Figure S6A), demonstrating that this TCF/LEF binding site is necessary for *LADLRM* activity (Figure 6A).

**FIGURE 7:**
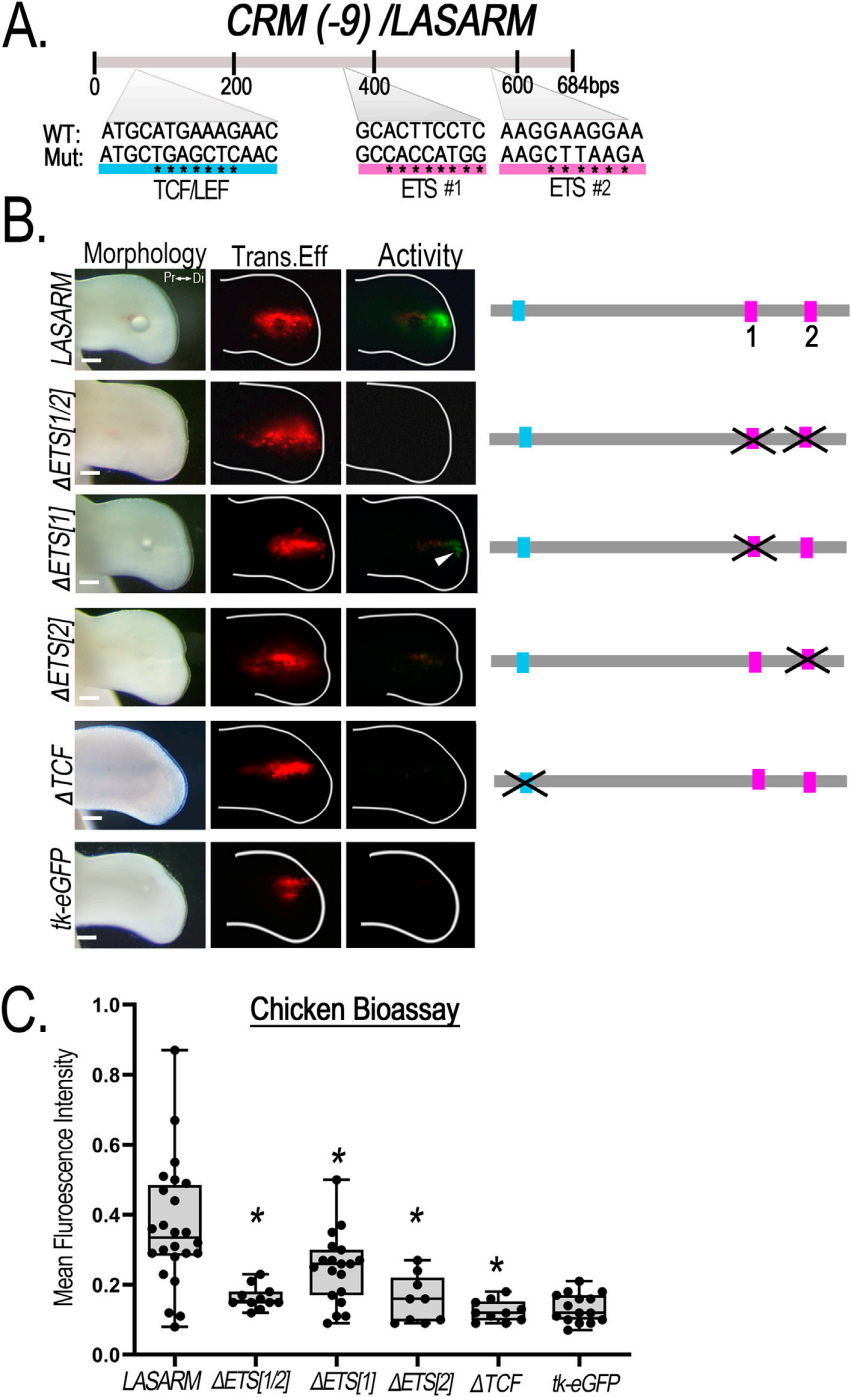
Disruption of putative ETS and TCF/LEF binding sites alters *CRM (-9)/LASARM* activity. **(A)** Schematic representation of the relative location of the two putative ETS (pink) and single TCF/LEF (blue) binding sites sequence in *LASARM*. Asterisks (*) indicate the nucleotide base pair substitutions performed to generate mutated (Mut) sequences compared to the wild type (WT). **(B)** Mutation of both ETS binding sites (ΔETS [1/2], n = 11) together abolishes *LASARM* activity. Disruption of ETS binding site 1 (ΔETS [1], n = 19) significantly reduces activity, while disruption of the single ETS binding site 2 (ΔETS[2], n = 9) or TCF/LEF binding site (ΔTCF, n = 10) abolishes *LASARM* activity to the level of the empty reporter vector (tk-eGFP, n = 15). **(C)** Boxplots depicting mean fluorescence intensity of the CRM-GFP (normalized to RFP) using Fiji software. One-way ANOVA followed by Dunnett’s multiple comparison test was performed using *LASARM* (n = 24) as the control, *p < 0.05. Abbreviations used: Pr (proximal), Di (distal). Scale bars: 250 µm.

*LASARM* possesses two conserved putative ETS binding sites, 155 bp apart, and one putative TCF/LEF binding site (Figure 7). Mutation of both ETS binding sites (ΔETS [1/2]) eliminated activity (Figure 7). Mutation of ETS binding site 1 (ΔETS[1]) significantly reduced activity (Figure 7) when compared to wildtype, suggesting that this site contributes to, but is not necessary for, *LASARM* activity. Disruption of either the ETS binding site 2 (ΔETS[2]) or the TCF/LEF binding site (ΔTCF) was sufficient to remove activity (Figure 7) as statistical analysis revealed activity was not significantly different from negative control (empty reporter vector, Supplementary Figure S6B). These results indicate that both the ETS binding site 2 and the TCF/LEF binding site are required for *LASARM* activity.

Binding of Tcf/Lef to both *LADLRM* and *LASARM* is supported by a preliminary report by Malkmus et al. (2024) showing associated binding of β-catenin (a co-factor in Tcf/Lef binding) by ChIP-seq to these CRMs (Supplementary Figure S6C). Unfortunately, we were unable to find published ChIP-seq data for embryonic mouse limbs for any of the ETS transcription factors to confirm binding to *LADLRM* or *LASARM*.

### 3.4 *ZRS* activity is unchanged in response to disrupted LHX2 binding sites

In *Lhx2/Lhx9* mutant mice, *Shh* expression is markedly reduced in the ZPA with mice exhibiting distal limb truncations (Tzchori et al., 2009). In chicken limbs, inhibition of *LHX2* function and morpholino knockdown of *LHX2* in the ZPA reduce *SHH* expression and aborts limb outgrowth (Rodriguez-Esteban et al., 1998; Watson et al., 2018). Furthermore, *LADLRM* and *LASARM* show intense activity in the distal posterior limb mesoderm overlapping the ZPA domain (Figures 3, 4), supporting a role for *Lhx2* in the expression and maintenance of *Shh*.

**FIGURE 8:**
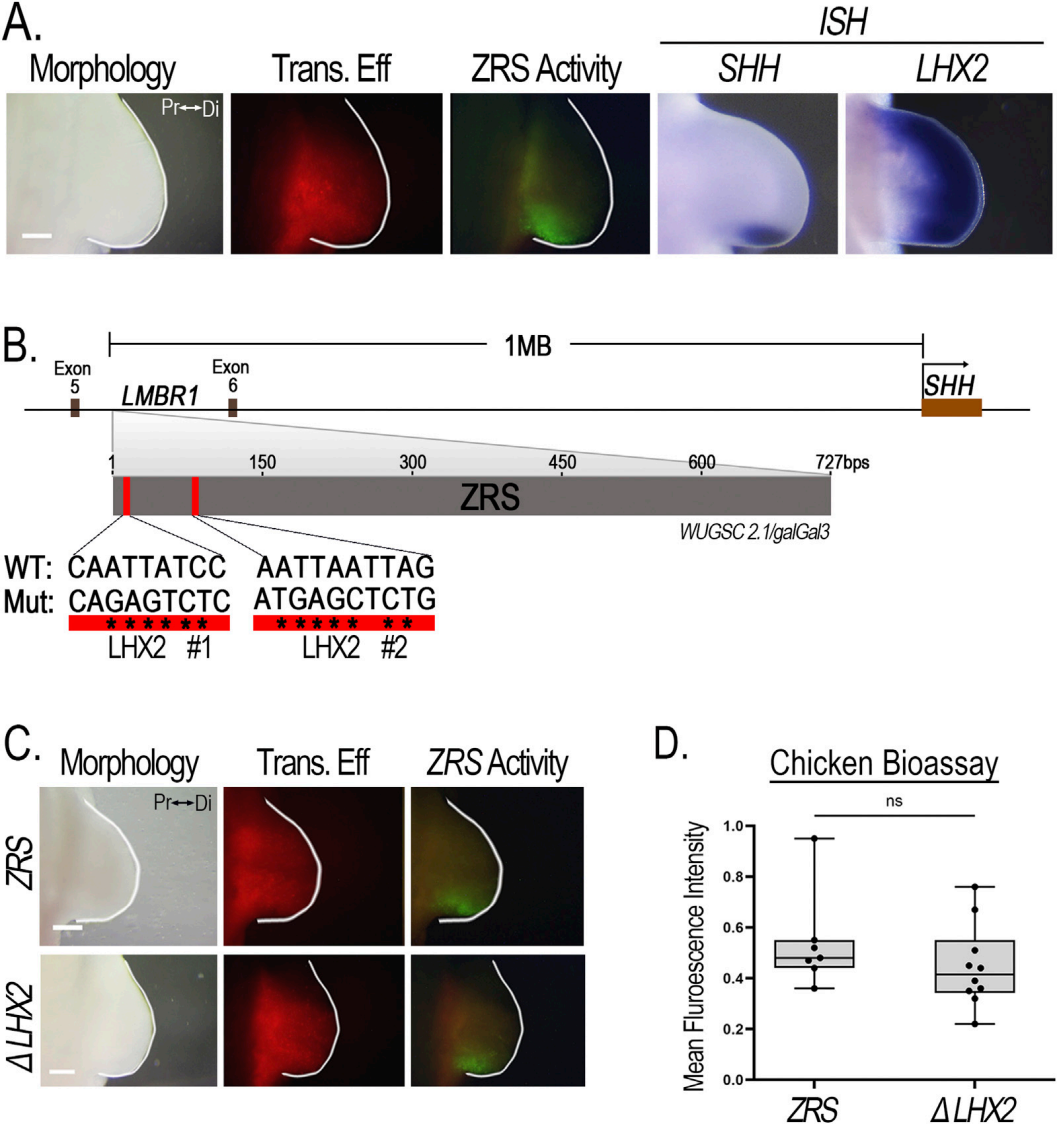
*ZRS* activity is not affected when LHX2 binding sites are disrupted. **(A)** *LHX2* expression overlaps the activity of the *ZRS* and *SHH* expression posteriorly in chicken limb buds. **(B)** Schematic diagram showing the *ZRS* situated between Exon 5 and 6 of the *LMBR1* gene, 1 Megabase (Mb) upstream of the *SHH* promoter. The *ZRS* contains two putative LHX2 binding sites (red rectangles). Asterisks (*) indicate the nucleotide base pair substitutions performed to generate mutated (Mut) sequences compared to the wild type (WT). **(C)** Disruption of the two LHX2 binding sites (ΔLHX2, n = 10) yields no change in *ZRS* activity compared to wildtype (*ZRS*, n = 7). **(D)** Boxplots depicting the mean fluorescence intensity of the *ZRS*-GFP-reporter (normalized to RFP) using Fiji software. Transfection efficiency (Trans. Eff) was assessed with a β-actin promoter-driven RFP plasmid. Student’s t-test (two-tailed, p < 0.05) was performed comparing the WT *ZRS* to the mutant. Abbreviations used: Pr (proximal), Di (distal), ns (non-significant). Scale bars: 250 µm.

The limb-specific *ZRS* enhancer regulates *Shh* expression (Lettice et al., 2002; Lettice et al., 2017). *Lhx2* expression overlaps *Shh* and *ZRS* activity in the ZPA (Figure 8A). The *ZRS* sequence contains two conserved putative Lhx2 binding sites that possess the core 5′-TAATTA-3′motif (Roberson et al., 1994), located 139 bp pairs from each other (Figure 8B). This provides a potential mechanism for direct regulation of *Shh* by Lhx2. To determine whether these two Lhx2 binding sites are required for *ZRS* activity, we simultaneously mutated both LHX2 binding sites (ΔLHX2) in the chicken. Surprisingly, the *ZRS* with mutant binding sites had no significant loss of activity (Figures 8C, D) indicating that Lhx2 does not regulate *Shh* expression through these two binding sites.

## 4 Discussion

Lhx2 modulates the delicate switch between the proliferation and differentiation of progenitor cells into their respective lineages by regulating the expression of pathway-specific genes during embryogenesis (Hsu et al., 2015; Li et al., 2022; Zibetti et al., 2019). For example, during brain development, Lhx2 via the Wnt/*B*-catenin pathway supports the proliferation and subsequent neurogenesis of cortical progenitors needed for appropriate cortex growth and differentiation; loss of *Lhx2* results in precocious initiation of neurogenesis and a smaller cortex (Bulchand et al., 2001; Hsu et al., 2015). In addition, Lhx2 exhibits pioneering transcription factor activity, establishing competence for gene expression by increasing chromatin accessibility and recruiting transcriptional proteins to developmental gene loci that regulate cell fate (Suresh et al., 2023; Zibetti et al., 2019). For instance, in the retina, Lhx2 remodels the chromatin of retinal progenitor cells in preparation for development and sustains their proliferation, with loss of *Lhx2* resulting in premature differentiation (Gordon et al., 2013; Zibetti et al., 2019).

In the limb, *Lhx2* is expressed in the distal mesoderm sub-adjacent to the apical ectodermal ridge (AER) and overlapping the zone of polarizing activity (ZPA) (Rodriguez-Esteban et al., 1998; Tzchori et al., 2009; Watson et al., 2018; Yokoyama et al., 2017). This sub-AER mesoderm is a region of undifferentiated progenitor cells that contributes to patterned limb outgrowth (Savage et al., 1993; Hara et al., 1998). Tzchori et al. (2009) suggested that Lhx2 (and Lhx9) function to integrate AER-Fgfs and ZPA-Shh signals that sustain progenitor cell populations in the distal limb during patterning and outgrowth. However, the mechanisms that induce and maintain Lhx2 to support these functions during limb development are unknown.

### 4.1 Identification of two *Lhx2*-associated regulatory modules with limb-specific activity

In this study, we identified two *cis*-regulatory modules (CRMs) within the *Lhx2* locus with limb-specific activity in the distal mesoderm coincident with *Lhx2* expression (Figures 3, 4). Functional enhancers of *Lhx2* have been identified for the midbrain and hindbrain (Lee et al., 2011); however, our study is the first to identify limb-specific *Lhx2* CRMs active in the limb. We named the CRMs based on their activity pattern, with *Lhx2*-associated distal Iimb regulatory module (*LADLRM*) exhibiting more broad activity extending about 500 μm from the distal tip with accentuated activity over the ZPA. The other CRM, *Lhx2*-associated sub-AER regulatory module (*LASARM*), has activity within the cells just beneath the AER (sub-AER). The intense activity of both *LADLRM* and *LASARM* in the posterior ZPA-related domain implies these CRMs work to ensure that *Lhx2-*expressing cells are kept responsive to AER and ZPA signals during proximodistal limb segment differentiation and anteroposterior patterning.

The AER expresses multiple Fgfs (*Fgf4*, *Fgf8*, *Fgf9*, and *Fgf17*) (Sun et al., 2002) and Wnts (*Wnt3*, *Wnt4*, *Wnt6*, and *Wnt7b*) (Geetha-Loganathan et al., 2008) that contribute to AER formation and maintenance. These factors also participate in the feedback loops that control limb outgrowth and patterning. Ets and Tcf/Lef transcription factors are part of the Fgf and Wnt signaling pathways, respectively. Ets transcription factors are recognized as regulators of proximodistal and anteroposterior limb patterning (Koyano-Nakagawa et al., 2022; Mao et al., 2009; Yamamoto-Shiraishi et al., 2014). For example, data suggest that the Etv4/5 transcription factors regulate anteroposterior patterning by localizing *Shh* expression to the ZPA. In their absence, ectopic anterior *Shh* is expressed, and radial polydactyly develops (Koyano-Nakagawa et al., 2022; Lettice et al., 2012; Mao et al., 2009; Zhang et al., 2009). Tcf/Lef transcription factors are also important regulators of AER development, limb outgrowth, and proximodistal patterning (Kawakami et al., 2001; Kengaku et al., 1998; ten Berge et al., 2008). For instance, *Lef1* ^−/−^ *Tcf1* ^−/−^ mutant mice fail to develop a functional AER and limb development is arrested (Galceran et al., 1999). The co-expression of several *Ets* and *Tcf/Lef* transcripts in *Lhx2*-expressing cells, the associated binding of β-catenin to both CRMs, and the importance of their predicted binding sites for both *LADLRM* and *LASARM* activity suggest that Fgfs and Wnts participate in the maintenance of *Lhx2* expression.

An unexpected finding in this study is the differential activity pattern of *LADLRM* and *LASARM* between the chicken and mouse. In chicken, *LADLRM* derived from either chicken or mouse is active in both the dorsal and ventral mesoderm, whereas in mice, the murine *LADLRM* is active only in the dorsal limb mesenchyme. These data suggest that mouse ventral mesoderm, but not chicken ventral mesoderm, is expressing a regulatory transcription factor that is inhibiting *LADLRM* activity. Similarly, the chicken *LASARM* has no dorsal-ventral bias in the chicken. However, the activity of the murine *LASARM* sequence in transgenic mice is accentuated ventrally, while posteriorly, adjacent to the ZPA, the activity is also expressed in the dorsal mesoderm. The suspected regulatory transcription factor in the mouse ventral limb mesoderm could also act to ventrally accentuate *LASARM* activity. Further work is needed to clarify the transcriptional differences between murine and chicken ventral limb bud mesoderm.

A probable explanation is the species-specific (chicken-mouse) differences in *Lhx2* and *Lhx9* expression. In chicken, *LHX9* is expressed only in the anterior mesoderm and does not play a role in the regulation of *SHH* (Watson et al., 2018). However, *LHX2* is expressed throughout the distal mesenchyme, extending the full anterior and posterior length of the limb, and is necessary for the maintenance of *SHH* expression (Watson et al., 2018). Moreover, repression of chicken *LHX2* expression stops limb outgrowth and halts AER formation (Rodriguez-Esteban et al., 1998). This suggests that in chicken *LHX2* may be the main LIM homeodomain transcription factor in the distal sub-AER mesoderm to regulate patterning and outgrowth, requiring *LADLRM* activity in both the dorsal and ventral mesoderm. In contrast in mice, *Lhx9* expression extends beyond the anterior mesoderm at the distal tip and into the posterior limb domain overlapping the ZPA. The redundant, overlapping *Lhx9* expression in the *Lhx2* domain may lessen the constraints for *Lhx2* to have both dorsal and ventral expression. Alternatively, mice may harbor another ventrally-active CRM to regulate the ventral expression of *Lhx2*.

### 4.2 *ZRS* activity is independent of LHX2 binding sites

Although *Lhx2* is required for *Shh* activity, and both *LADLRM* and *LASARM* show accentuated posterior activity associated with the ZPA, our data indicate that predicted LHX2 binding sites are not necessary for the activity of the *ZRS*, the Shh enhancer, in an isolated reporter construct. Similarly, preliminary data from Bower et al. (2024) found that disruption of Lhx2 binding sites within a *ZRS*-reporter construct did not alter activity; however, when these same sites are removed from the endogenous *ZRS* in mice, limb truncation defects occurred indistinguishable from the complete loss of Shh. They suggest that these binding sites are necessary for mediating long-range enhancer activation (Bower et al., 2024). Collectively these data suggest a role for Lhx2 in tethering the *ZRS* to its promoter region (or promoting their interaction) and a rationale for the accentuated ZPA-related activity of the *Lhx2* CRMs. Alternatively, accentuated ZPA-related *Lhx2* expression could increase chromatin accessibility (Zibetti et al., 2019) and accentuate ZPA-related *Shh* transcription compared to other sites lacking Lhx2.

*Lhx2* could also regulate *Shh* expression independent of the *ZRS*. Shh co-receptors Cdon and Gas1 mediate Shh signaling and regulate limb patterning and digit specification, as mutants display zeugopod and autopod defects (Allen et al., 2007; Allen et al., 2011; Echevarría-andino et al., 2023). In mouse retinal progenitor cells, *Lhx2* binds to the Cdon and Gas1 loci to support activation of the *Shh* (Li et al., 2022) and likewise may do so in the limb.

### 4.3 Summary

In summary, we discovered two *Lhx2*-associated *cis*-regulatory modules, *LADLRM* and *LASARM,* within the *Lhx2* locus that have limb-specific activity overlapping *Lhx2* expression. Several *Ets* and *Tcf/Lef* members co-express with *Lhx2*. Moreover, Ets transcription factor binding sites contribute to *LADLRM* activity and are necessary for *LASARM* activity. Additionally, Tcf/Lef transcription factor binding sites are essential for both the activity of *LADLRM* and *LASARM.* Together, these data suggest that Fgf and Wnt signaling pathways participate in regulating *Lhx2* expression in the distal limb mesoderm.

## Data Availability

The original contributions presented in the study are included in the article/Supplementary Material, further inquiries can be directed to the corresponding author.
