# Supplementary material for: Evidence for Fgf and Wnt regulation of Lhx2 during limb development via two limb-specific Lhx2-associated cis-regulatory modules
Source: Front Cell Dev Biol. 2025 Feb 20;13:1552716. doi: 10.3389/fcell.2025.1552716 (PMC11882541; doi:10.3389/fcell.2025.1552716)
Supplement: Supplementary file 1 [file DataSheet1.pdf]

Supplementary Material

1 Supplementary Figures

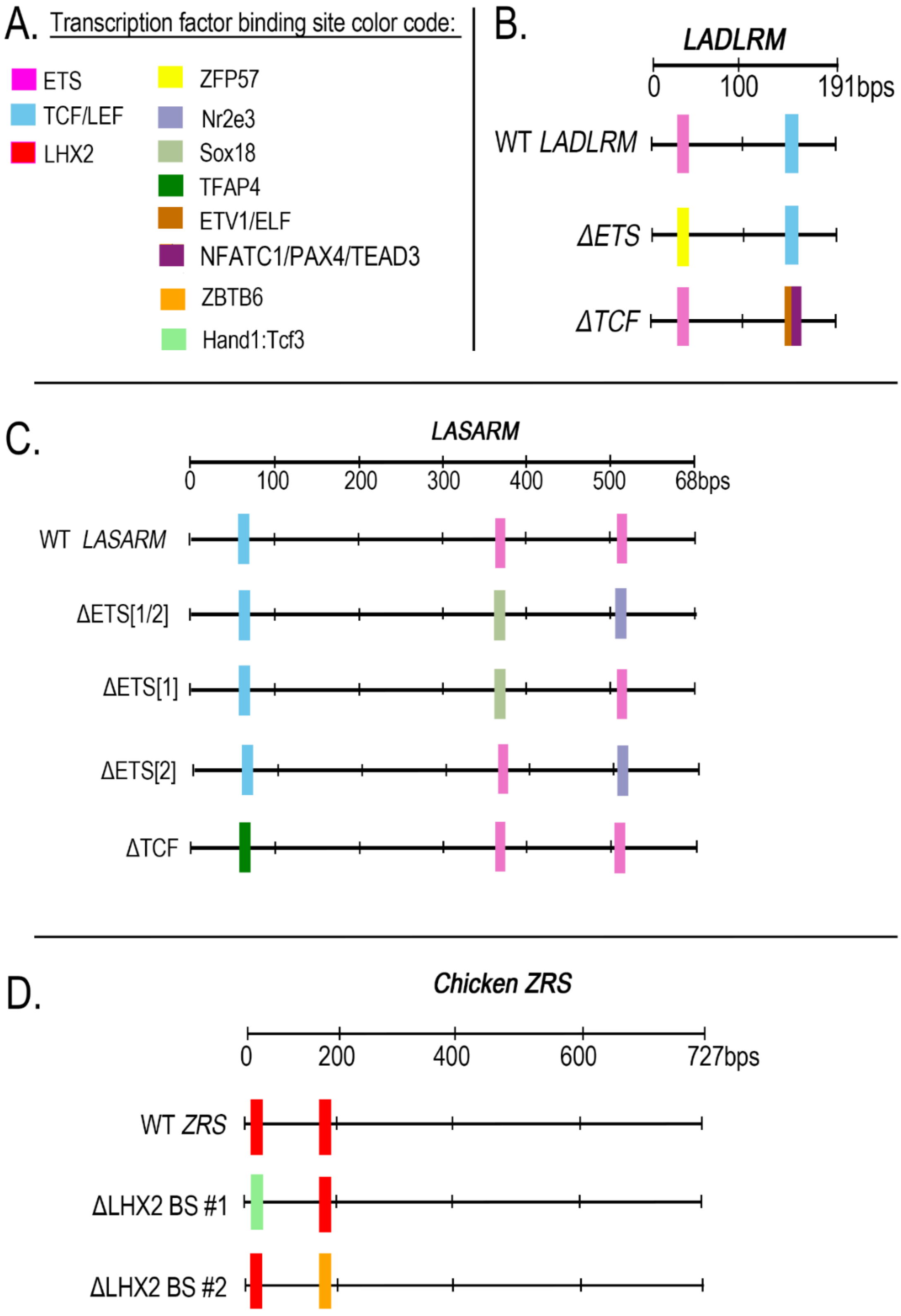

Supplemental Figure S1. Transcription factor binding sites analysis of wild-type and mutated chicken sequences of *LADLRM*, *LASARM*, and chicken *ZRS*. A) Color code for putative and introduced transcription factor binding sites within *LADLRM*, *LASARM*, and the *ZRS*. Schematic diagram was modified from CiiiDER transcription factor binding site prediction and visualization software.

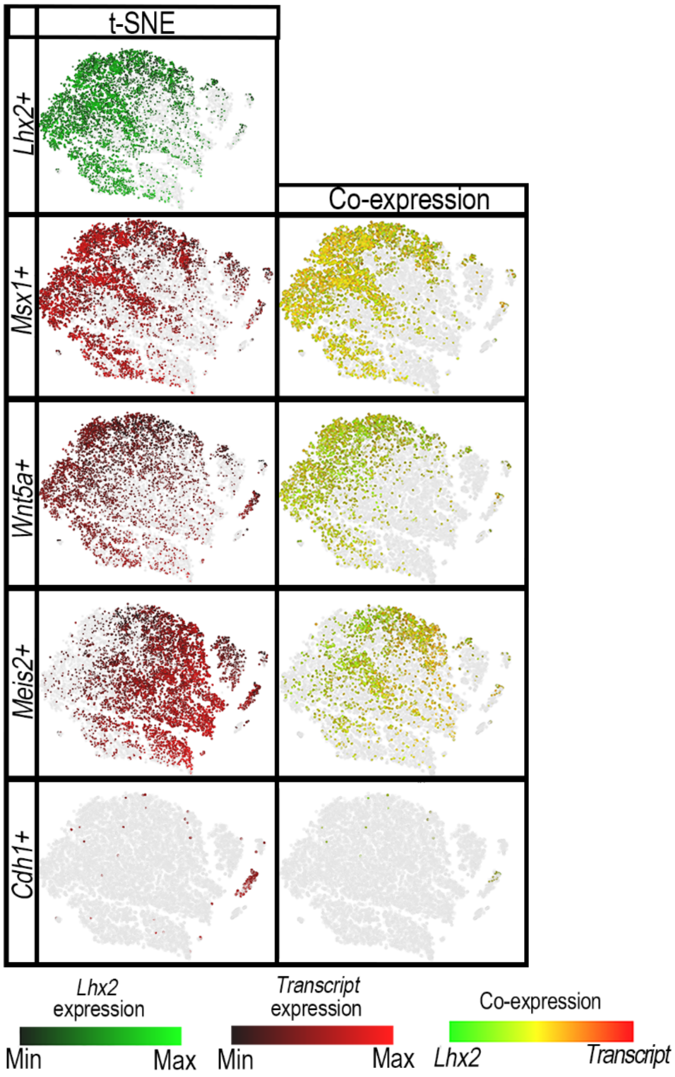

Supplemental Figure S2. t-SNE plots showing *Lhx2, Msx1, Wnt5a,* and *E-cadherin* transcripts expressed in E11 mouse limb cells. *Msx1+* and *Wnt5a+* cells mark the distal mesenchyme*,* while *Meis2+* cells mark the proximal mesenchyme. Less than 1% of the cells available for analysis were of ectodermal origin (*E-cadherin: Cdh1+*) confirming their mesodermal origin.

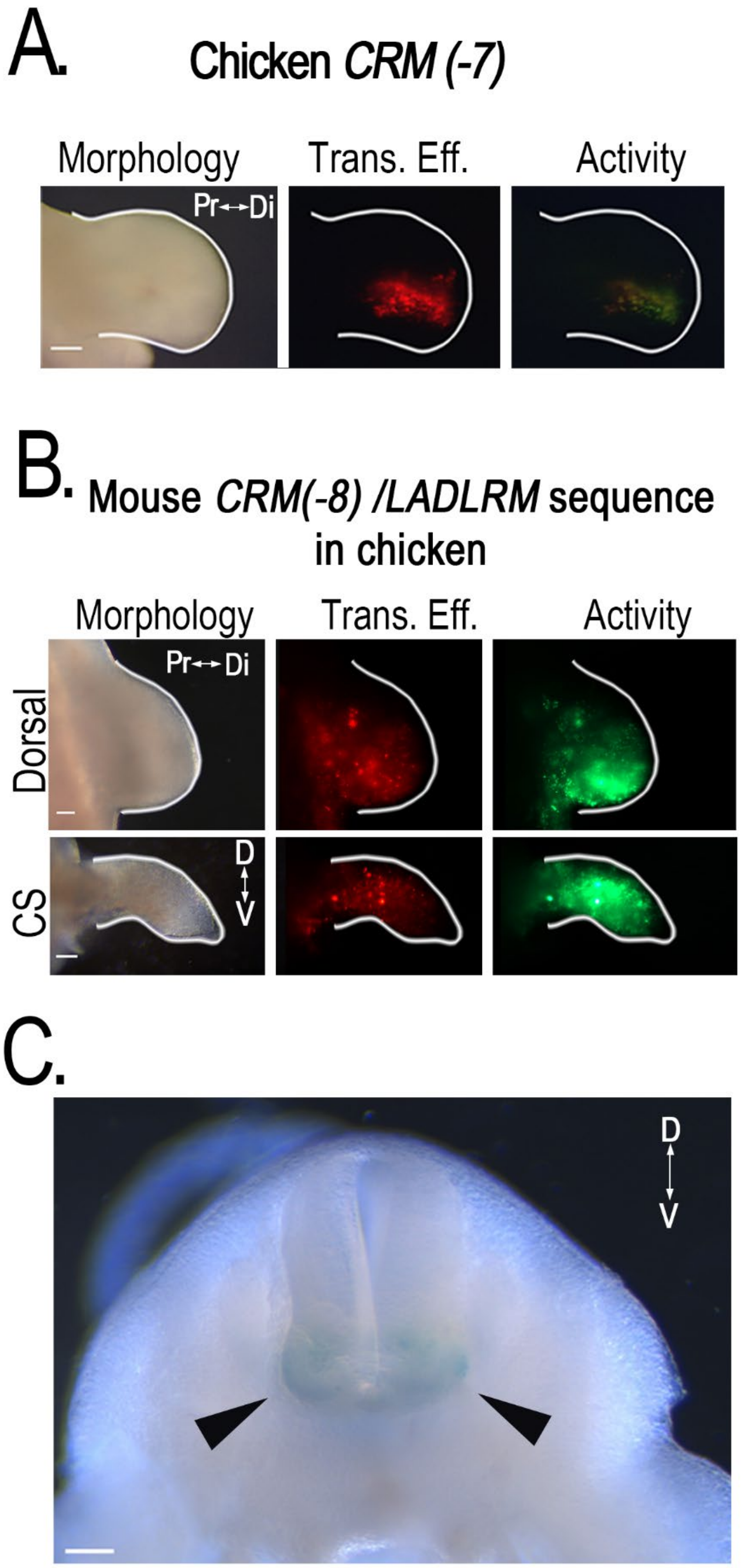
A.
Chicken *CRM (-7)*
Morphology
Trans. Eff.
Activity
Pr↔Di
B.
Mouse *CRM(-8) /LADLRM* sequence in chicken
Morphology
Trans. Eff.
Activity
Dorsal
CS
Pr↔Di
D
V
C.
D
V

Supplemental Figure S3. A) The chicken *CRM (*-7*)* sequence exhibited weak, inconsistent activity (n=10/45) in the distal limb of Hamburger Hamilton (HH) stage 25 chicken embryos. B) Mouse *CRM (-8)/LADLRM* sequence in chicken bioassay shows no dorsoventral bias activity in chicken limb mesoderm following Targeted Regional Electroporation in the presumptive limb bud (n=6). Note the accentuated activity in the posterior mesoderm. Longitudinal cross-sections (CS) of the forelimb showed *CRM (-8)/ LADLRM* activity in both the dorsal and ventral mesoderm. Transfection efficiency (Trans. Eff) was determined with a β-actin promoter-driven RFP plasmid. C) Cross section of the neural tube in transgenic mice shows weak X-gal staining in the ventral basal plate (black arrows). Scale bars: 250 µm.

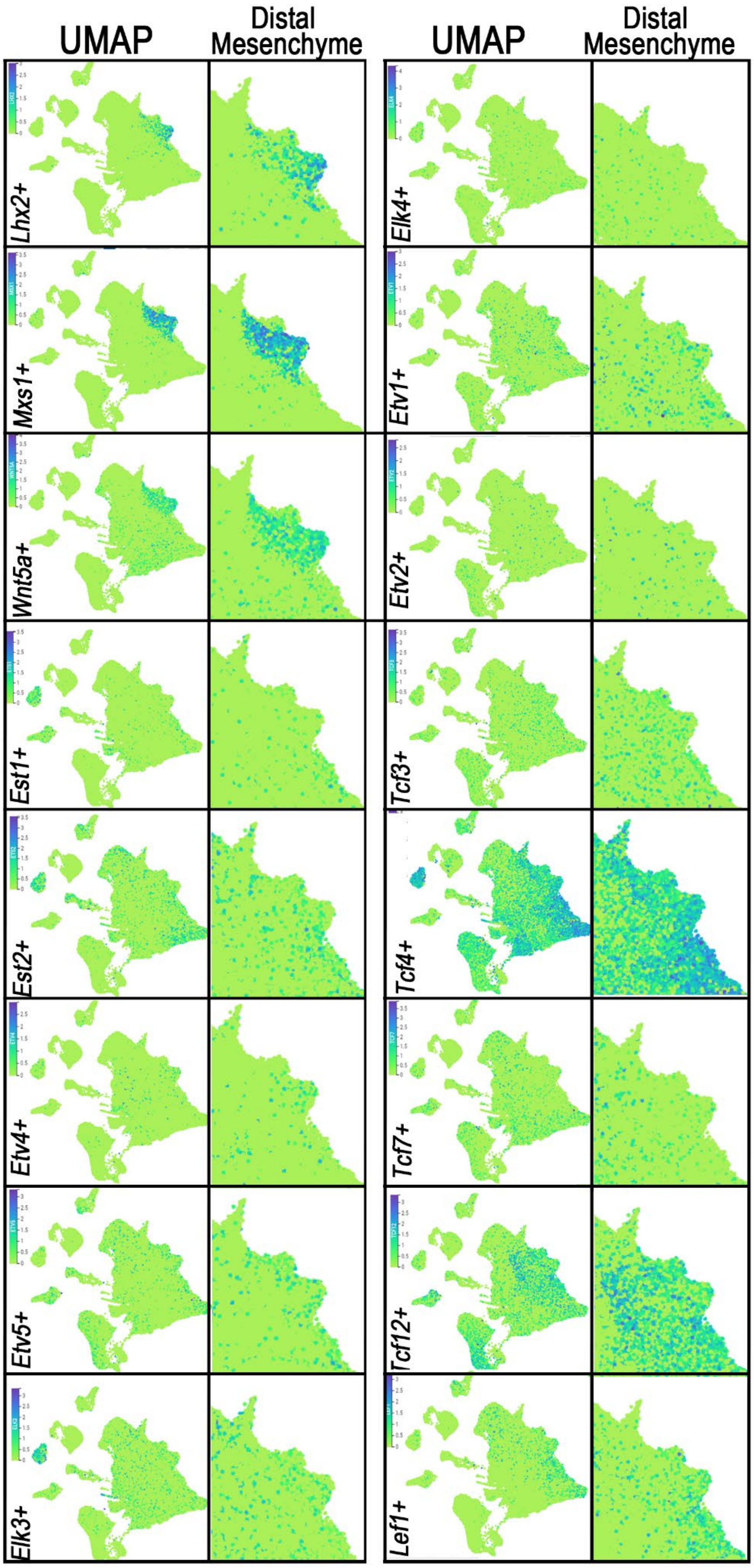
UMAP
Distal Mesenchyme
UMAP
Distal Mesenchyme
Lhx2+
Mxs1+
Wnt5a+
Est1+
Est2+
Etv4+
Etv5+
Elk3+
Elk4+
Etv1+
Etv2+
Tcf3+
Tcf4+
Tcf7+
Tcf12+
Lef1+

Supplemental Figure S4. UMAP cell plots showing colocalization (green to blue) of *Ets* and *Tcf/Lef* transcripts in the distal limb mesenchyme of embryonic human limbs. scRNA-seq data of embryonic human limbs, post-week conception (PWC) 5.6 (Zhang et al., 2023) was analyzed using CELLXGENE open-source software (https://limb-dev.cellgeni.sanger.ac.uk/) and annotated in Adobe Photoshop Version 2024.

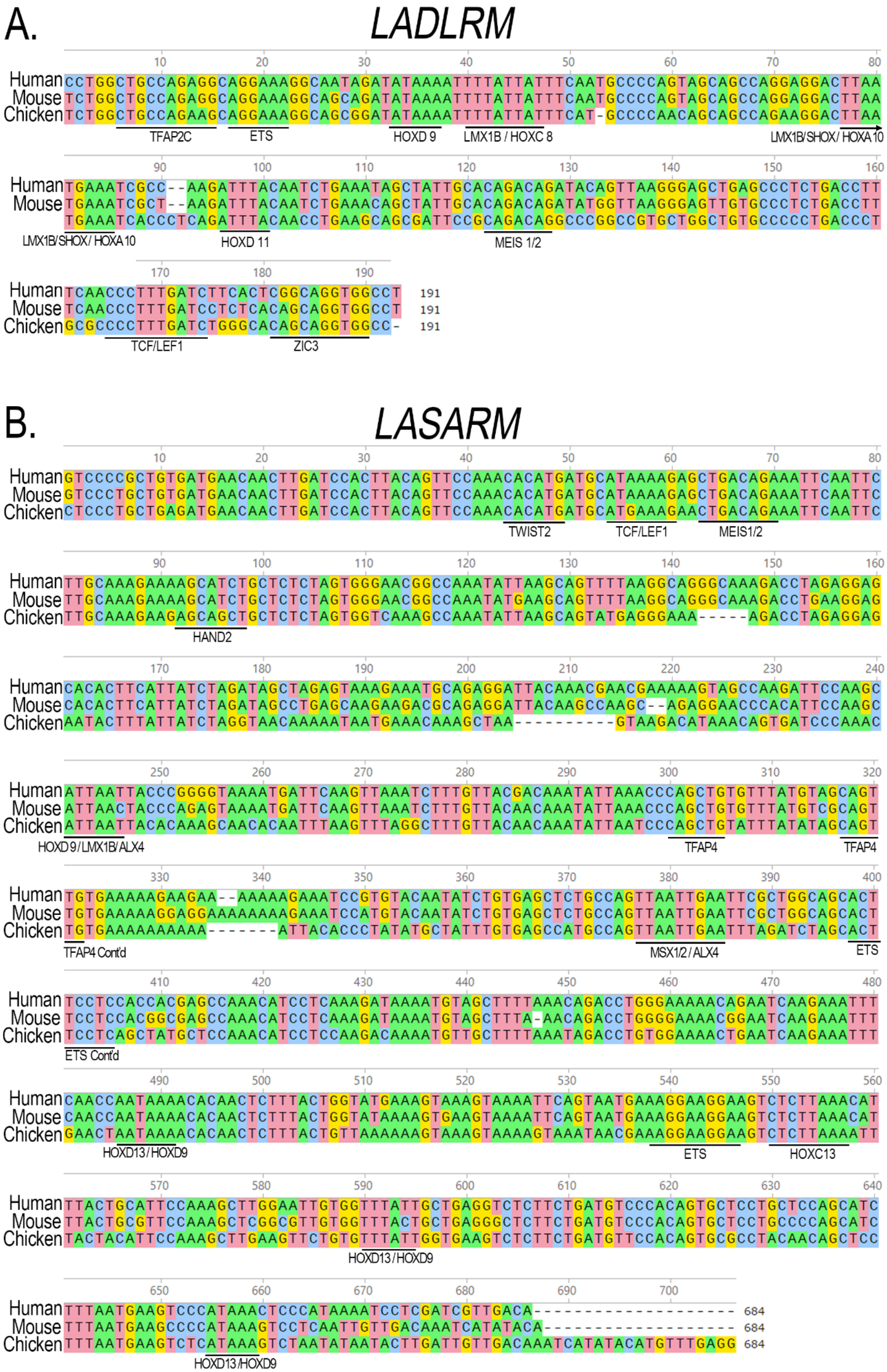
A.
LADLRM
Human
Mouse
Chicken
TFAP2C
ETS
HOXD 9
LMX1B / HOXC 8
LMX1B/SHOX/ HOXA 10
HOXD 11
MEIS 1/2
TCF/LEF1
ZIC3
B.
LASARM
TWIST2
TCF/LEF1
MEIS1/2
HAND2
HOXD9/LMX1B/ALX4
TFAP4
TFAP4 Cont'd
MSX1/2 / ALX4
ETS
ETS Cont'd
HOXD13 / HOXD9
HOXC13

Supplemental Figure S5. Clustal Omega alignment of the human, mouse, and chicken *LADLRM* and *LASARM* sequences. Identity between species – *LADLRM:* Chicken–Mouse (82%), Chicken-Human (80%), Mouse-Human (94%); *LASARM:* Chicken-Mouse (78%), Chicken-Human (81%), Mouse-Human (90%). Binding sites of transcription factors commonly expressed in the limb with prediction binding scores of p value: $\leq 10^{-2}$ are underlined in black.

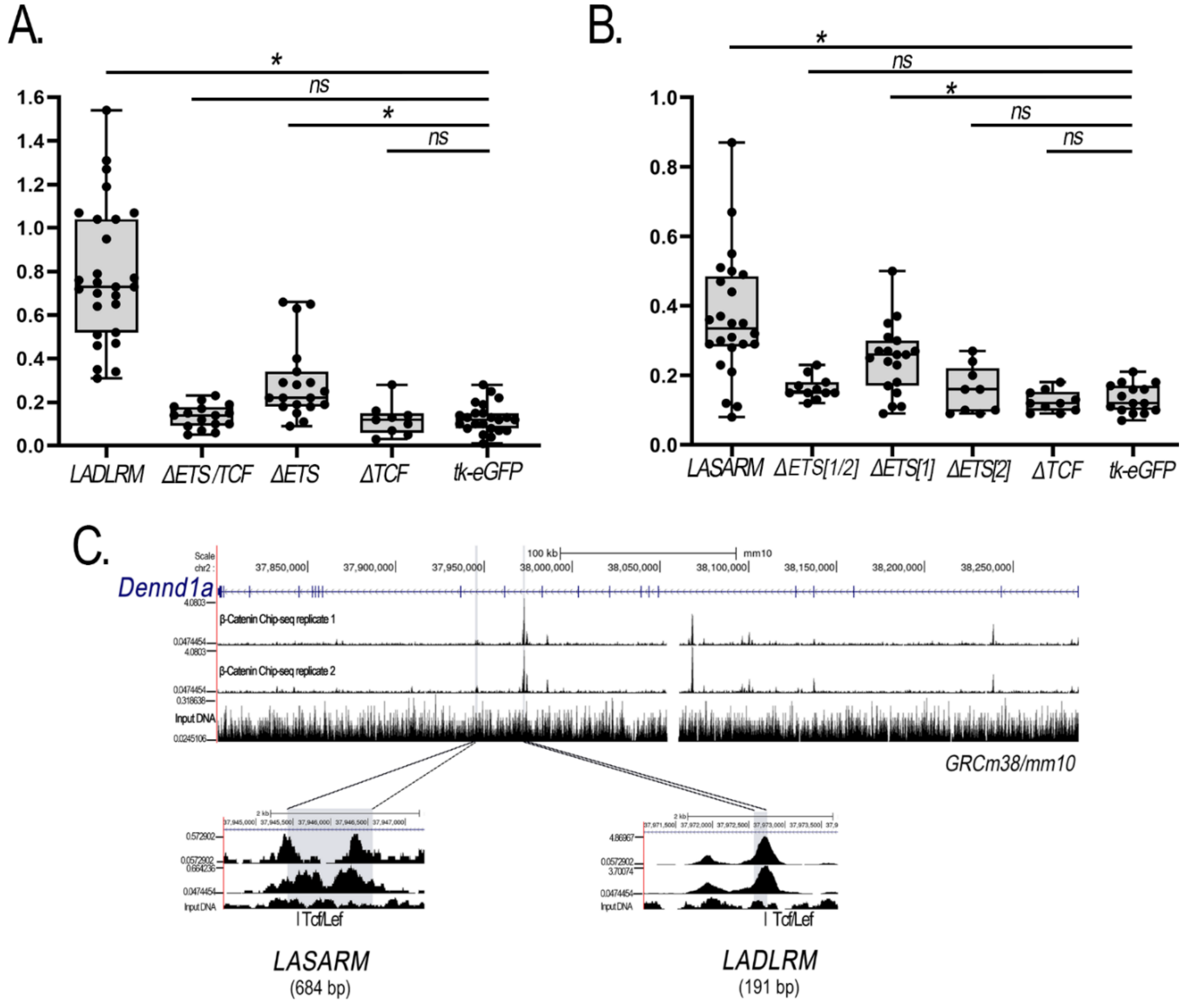

Supplemental Figure S6. Boxplots depicting the mean fluorescence intensity using Fiji software. Dunnett's test, compared to negative (tk-eGFP) control (n=28), using one-way ANOVA * $p<0.05$. A) For *LADLRM*, disruption of putative ETS and TCF binding site (ΔETS/TCF, n=16) together, or disruption of the TCF/LEF binding site (ΔTCF, n=9) alone, ablates *LADLRM* activity and is non-significant when compared to negative control. B) For *LASARM*, mutation of the ETS binding site 2 (ΔETS [2], n=19), or the TCF/LEF binding site (ΔTCF, n=9) decreases *LADLRM* activity and is non-significant when compared to negative control. C). Alignment of murine *β*-Catenin Chip-seq data for E10.5 limb buds (Malkmus et al., 2024) reveals binding of *β*-Catenin to the Tcf/Lef binding sites in *LADLRM* and *LASARM*. The region of *LADLRM* and *LASARM* is highlighted in gray.

References for Supplemental Material

Gearing, L. J., Cumming, H. E., Chapman, R., Finkel, A. M., Woodhouse, I. B., Luu, K., Gould, J. A., Forster, S. C., & Hertzog, P. J. (2019). CiIIder: A tool for predicting and analysing transcription factor binding sites. *PLoS ONE*, *14*(9), 1–12. https://doi.org/10.1371/journal.pone.0215495

He, P., Williams, B. A., Trout, D., Marinov, G. K., Amrhein, H., Berghella, L., Goh, S. T., Plajzer-Frick, I., Afzal, V., Pennacchio, L. A., Dickel, D. E., Visel, A., Ren, B., Hardison, R. C., Zhang, Y., & Wold, B. J. (2020). The changing mouse embryo transcriptome at whole tissue and single-cell resolution. In *Nature* (Vol. 583, Issue 7818). Springer US. https://doi.org/10.1038/s41586-020-2536-x

Malkmus, J., Morabito, A., Lopez-Delisle, L., Esteban, L. A., Mayran, A., Zuniga, A., Sharpe, J., Zeller, R., & Sheth, R. (2024). WNT signaling coordinately controls mouse limb bud outgrowth and establishment of the digit-interdigit pattern Jonas. *BioRxiv*, *12*(24), 629665. https://doi.org/10.1101/2024.12.25.629665

Zhang, B., He, P., Lawrence, J. E. G., Wang, S., Tuck, E., Williams, B. A., Roberts, K., Kleshchevnikov, V., Mamanova, L., Bolt, L., Polanski, K., Li, T., Elmentaite, R., Fasouli, E. S., Prete, M., He, X., Yayon, N., Fu, Y., Yang, H., … Teichmann, S. A. (2023). A human embryonic limb cell atlas resolved in space and time. *Nature*, *635*(809), 668–678. https://doi.org/10.1038/s41586-023-06806-x
